# Aqueous Extract of Descuraniae Semen Attenuates Lipopolysaccharide-Induced Inflammation and Apoptosis by Regulating the Proteasomal Degradation and IRE1α-Dependent Unfolded Protein Response in A549 Cells

**DOI:** 10.3389/fimmu.2022.916102

**Published:** 2022-06-24

**Authors:** Po-Chun Hsieh, Chung-Kan Peng, Guan-Ting Liu, Chan-Yen Kuo, I-Shiang Tzeng, Ming-Chieh Wang, Chou-Chin Lan, Kun-Lun Huang

**Affiliations:** ^1^Department of Chinese Medicine, Taipei Tzu Chi Hospital, Buddhist Tzu Chi Medical Foundation, New Taipei City, Taiwan; ^2^Graduate Institute of Medical Sciences, National Defense Medical Center, Taipei, Taiwan; ^3^Division of Pulmonary and Critical Care Medicine, Department of Internal Medicine, Tri-Service General Hospital, Taipei, Taiwan; ^4^Division of Pulmonary Medicine, Taipei Tzu Chi Hospital, Buddhist Tzu Chi Medical Foundation, New Taipei City, Taiwan; ^5^Department of Research, Taipei Tzu Chi Hospital, Buddhist Tzu Chi Medical Foundation, New Taipei City, Taiwan; ^6^Department of Pharmacy, Taipei Tzu Chi Hospital, Buddhist Tzu Chi Medical Foundation, New Taipei City, Taiwan; ^7^School of Medicine, Tzu Chi University, Hualien, Taiwan

**Keywords:** acute lung injury, lipopolysaccharide, Descuraniae semen, unfolded protein response, IRE1α, inflammation, apoptosis

## Abstract

**Background:**

Lipopolysaccharide (LPS)-induced acute lung injury (ALI) induces endoplasmic reticulum stress, unfolded protein response (UPR), apoptosis, and inflammation. Inositol-requiring enzyme 1 (IRE1)-α is important for adaptive and apoptotic UPR determination during ER stress. The aqueous extract of Descuraniae Semen (AEDS) is reported to be a safe and effective herb for the treatment of pulmonary edema as it shows anti-inflammatory activities.

**Methods:**

We investigated the effects of AEDS on LPS-induced ALI in A549 cells with respect to the regulation of IRE1α-dependent UPR, proteasomal degradation, mitochondrial membrane potential (MtMP), inflammation, and apoptosis.

**Results:**

AEDS attenuated ER stress by regulating the proteasomal degradation. LPS induced ER stress [binding immunoglobulin protein (BiP), phosphorylated IRE1α, sliced X-box binding protein 1 [XBP1s], phosphorylated cJUN NH2-terminal kinase (pJNK), B-cell lymphoma (Bcl)-2-associated X (Bax), Bcl-2], inflammation (nucleus factor-kappa B (NF-κB) p65 nuclear translocation, nucleus NF-κB, pro-inflammatory cytokines] and apoptosis [C/EBP homologous protein (CHOP), cytochrome c, caspase-8, and caspase-6, and TUNEL] were significantly attenuated by AEDS treatment in A549 cells. AEDS prevents LPS-induced decreased expression of MtMP in A549 cells.

**Conclusions:**

AEDS attenuated LPS-induced inflammation and apoptosis by regulating proteasomal degradation, promoting IRE1α-dependent adaptive UPR, and inhibiting IRE1α-dependent apoptotic UPR. Moreover, IRE1α-dependent UPR plays a pivotal role in the mechanisms of LPS-induced ALI. Based on these findings, AEDS is suggested as a potential therapeutic option for treating patients with ALI.

## Introduction

Acute lung injury (ALI) is a common cause of respiratory failure and is characterized by the acute onset of non-cardiogenic pulmonary edema, inflammatory cell infiltration, and impaired gas exchange, resulting in severe hypoxemia, and dyspnea that require mechanical ventilation (1, 2). Sepsis is the leading cause of ALI and accounts for about 33% of the etiology of ALI (3). Sepsis leads to systemic inflammatory responses and increased levels of the pro-inflammatory cytokine, such as the tumor necrosis factor α (TNF-α), interleukin (IL)-1β, IL-6, and IL-8 resulting in diffuse alveolar epithelial-endothelial barrier damage (1). The sepsis-associated ALI mortality rate is estimated to be 27-37% (4). Despite ongoing clinical trials, there are no consistently effective drugs for ALI treatment (2). Therefore, it is vital to develop effective therapies for sepsis-associated ALI.

Various stimuli, such as infection and hypoxia, can disrupt the endoplasmic reticulum (ER) function and accumulate unfolded proteins, leading to ER stress and stimulating the unfolded protein response (UPR) (5). UPR maintains ER functions and protein homeostasis (5). Inositol-requiring enzyme 1 (IRE1)-α is an ER transmembrane sensor. Upon ER stress, binding immunoglobulin protein (BiP) dissociates from IRE1α, promoting its autophosphorylation and dimerization, and activating the UPR (5). Phosphorylated IRE1α (pIRE1α) activates the X-box binding protein 1 (XBP1s) and ER-associated degradation (ERAD) to alleviate the elevated ER stress and promote cell survival, which is considered an adaptive UPR (6). During prolonged or excessive ER stress, pIRE1α activates the cJUN NH2-terminal kinase (JNK) and CCAAT-enhancer-binding protein homologous protein (CHOP) pathways to promote apoptosis, which is considered an apoptotic UPR (6). IRE1α is considered to be a master regulator of adaptive and apoptotic UPR determination during ER stress (6). ER and mitochondria interact in many physiological functions, including Ca^2+^ signaling, lipid transport, energy metabolism, and cell survival, through the close ER-mitochondria interface (7). ER-mitochondria interface is also involved in the coordination of Ca^2+^ transfer, autophagy, and inflammasome formation (8). An adaptive UPR maintains ER homeostasis and regulates dynamic interactions between ER, mitochondria, and autophagy to support ER function (9). Previous studies demonstrated that lipopolysaccharide (LPS)-induced ALI leads to ER stress, UPR, apoptosis, and inflammation, which are attenuated by 4-phenylbutyrate, an ER stress inhibitor (10–12). However, the regulatory mechanisms underlying IRE1α-dependent UPR cascades in LPS-induce ALI remain largely unknown.

Descuraniae Semen (DS) is the dried seed of *Descurainia sophia* L. Webb ex Prantl (13). DS is an effective and safe herb that is used in complementary and alternative medicine for the treatment of cough, asthma, and pulmonary edema (14). In clinical practice, DS is used as the aqueous extract of DS (AEDS). DS exerts anti-inflammatory and antioxidative effects (15, 16). Compared to the conventional treatment strategies for ALI, AEDS combined with conventional treatment strategies showed significantly better improvements in pulmonary vascular permeability and decreases in lung water after six days, with an overall lower mechanical ventilation days and mortality rate (17, 18). Zhang et al. reported that AEDS treatment in an LPS-induced ALI rat model decreased the pulmonary edema and inflammation, while increasing the expression levels of aquaporin 5 in lung tissues (19). However, the molecule mechanisms of AEDS in LPS-induced ALI remain unclear.

This study aimed to investigate the effects of AEDS on LPS-induced ALI in A549 cells with respect to the regulation of IRE1α-dependent UPR, inflammation, and apoptosis.

## Materials and Methods

### Reagents

The concentrated aqueous extract power of DS (Ting Li Zi, K850, #421312802) was provided by Ko Da Pharmaceutical Co., Ltd. (Taoyuan, Taiwan). One gram of the concentrated AEDS was derived from the 5 g of DS. LPS was purchased from Sigma-Aldrich (St. Louis, MO, USA).

### Antibodies

Antibodies against mouse, anti-rabbit IgG-conjugated horseradish peroxidase, BiP, caspase-6, caspase-8, CHOP, cytochrome c (Cyt C), B-cell lymphoma 2 (Bcl-2), Bcl-2-associated X (Bax), and activating transcription factor 6 (ATF6) were purchased from ABclonal (Woburn, Massachusetts, USA). Antibodies against β-actin, cleaved caspase-3 (cCaspase-3), and p-JNK were purchased from Cell Signaling Technology (Danvers, MA, USA). Antibodies against activating transcription factor 4 (ATF4) was purchased from Abcam (Cambridge, UK).

### Chemicals

TNF-α, IL-1β, IL-6, and IL-8 enzyme-linked immunosorbent assay (ELISA) kit was purchased from ABclonal (Woburn, MA, USA). Fetal bovine serum (FBS) and Dulbecco’s modified Eagle’s medium (DMEM) were purchased from Gibco-BRL Life Technologies (Grand Island, NY, USA). Cell counting kit-8 (CCK-8) was purchased from Dojindo (Kumamoto, Japan). JC-1 mitochondrial membrane potential (MtMP) assay kits and TUNEL apoptosis detection kit were purchased from Abcam (Cambridge, MA, USA). Proteasome inhibitor (MG132) was purchased from Sigma-Aldrich (St Louis, MO, USA).

### Cell Culture

Human lung epithelial cell line (A549) was purchased from the American Type Culture Collection (Manassas, VA, USA). Cells were cultured in 5% CO_2_ in DMEM supplemented with 10% FBS and 100 units/mL penicillin/streptomycin at 37°C.

### Experimental Protocol

**Figure 1:**
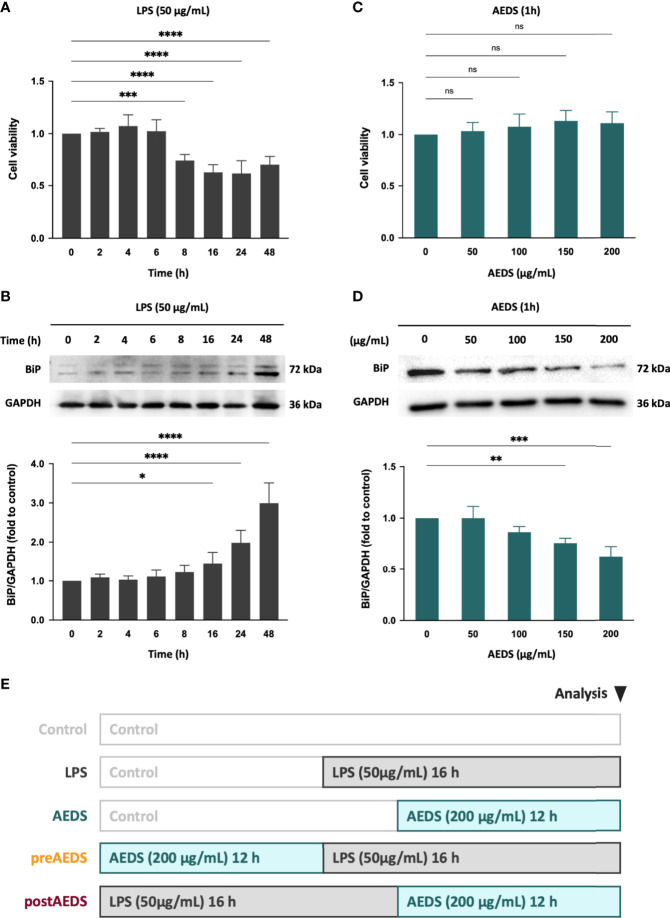
Effects of lipopolysaccharide (LPS) and the aqueous extract of Descuraniae Semen (AEDS) on cell viability and endoplasmic reticulum (ER) stress in A549 cells. **(A)** LPS (50 µg/mL) decreased the cell viability in a time-dependent manner after 8 h of induction. **(B)** LPS (50 µg/mL) increased the binding immunoglobulin protein (BiP) expression levels in a time-dependent manner after 16 h of induction. **(C)** AEDS exhibited no effects on the cell viability in a dose-dependent manner in 1 h **(D)** AEDS decreased the BiP expression levels in a dose-dependent manner in 1 h **(E)** Experimental protocols with five experiment groups: Control, LPS, AEDS, AEDS pre-treatment followed by LPS induction (preAEDS), and LPS induction followed by AEDS treatment (postAEDS) groups. Cell counting-kit (CCK)-8 assay was used to detect the cell viability. All data are expressed as the mean ± standard deviation (SD). All p-values were calculated using one-way analysis of variance followed by Tukey’s *post-hoc* test. The experiment was performed five times in duplicate. *p < 0.05, **p < 0.01, ***p < 0.001, ****p < 0.0001. ns, not significant.

Concentrated AEDS was dissolved in DMEM to a final concentration of 200 µg/mL. LPS was dissolved in DMEM to a final concentration of 50 µg/mL. A549 cells were seeded in a 6-well plate at a density of 1 × 10^6^ cells/well for 24 h. The medium was then replaced with a new basal medium and cultured under the conditions of five experimental groups: control (with DMEM), LPS, AEDS, AEDS pre-treatment followed by LPS induction (preAEDS), and LPS induction followed by AEDS treatment (postAEDS) groups (**Figure 1E**). The cells were then harvested and extracted.

### Cell Viability Assay

CCK-8 assay was used to detect the cell viability according to the manufacturer’s instructions (20). CCK-8 contains Dojindo’s highly water-soluble tetrazolium salt (WST-8), which can be reduced by dehydrogenases in cells and transform into orange colored WST-8 formazan. The amount of the generated WST-8 formazan dye directly proportional to the number of living cells. A549 cells were seeded at a density of 2 × 10^4^ cells/well of a 96-well plate and allowed to adhere overnight. Next, 10 μL of the CCK-8 solution was added to each well of the plate and incubated with the indicated concentrations of AEDS and LPS at 37°C with 5% CO2 for 2 h. Absorbance was measured at 450 nm using an Infinite 200 PRO microplate reader (Tecan, Männedorf, Switzerland).

### Western Blotting Analysis

We collected the cells and lysed them in cold radioimmunoprecipitation assay (RIPA) buffer containing protein inhibitors. Proteins were resolved on sodium dodecyl sulfate-polyacrylamide gel and transferred to immobilon polyvinyl difluoride membranes. Western blotting was performed using specific primary and horseradish peroxidase-conjugated secondary antibodies. Enhanced chemiluminescence was used to measure the peroxidase activity. Bio-Rad ChemiDoc XRS+ (Bio-Rad, Hercules, CA, USA) was used to analyze the intensities of the reactive bands.

### Proteasome Inhibitor MG132

MG132 is a potent, reversible, and cell-permeable proteasome inhibitor. MG132 effectively blocks the proteolytic activity of the 26S proteasome complex (21). We treated the cells with AEDS for 1 h, proteasome inhibitor MG132 (10 μM) alone for 6 h or pretreated them with MG132 followed by stimulation with AEDS. The cells were collected and lysed in cold RIPA buffer with the protein inhibitor and stored at –80°C until use.

### Mitochondrial Membrane Potential Assay

A549 cells (2 × 10^4^ cells) were seeded in each well of a 96-well plate and allowed to adhere overnight. The cells were cultured under the conditions of the five experimental groups. MtMP was measured using a JC-1 MtMP assay kit.

### TUNEL Apoptosis Assay

A549 cells (2 × 10^4^ cells) were seeded in 4-well multi-chamber culture slides (SPL, Gyeonggi-do, Korea) and cultured for 24 h. After treatment, the cells were fixed with 4% paraformaldehyde for 10 min at room temperature. Triton X-100 (0.2% solution) was used to permeabilize the cells for 1 min after washing thrice with phosphate-buffered saline (PBS). According to the manufacturer’s instructions, the cells were blocked in 3% FBS (diluted in 1% bovine serum albumin (BSA) in PBS) for 30 min and washed thrice with PBS before incubation with the terminal deoxynucleotidyl transferase dUTP nick and labeling (TUNEL) apoptosis detection Kit. The slides were examined using a LEICA DM6000 B inverted fluorescent microscope (Leica, Wetzlar, Germany).

### Immunofluorescence Analysis

A549 cells (2 × 10^4^ cells) were seeded in each well of a 4-well multi-chambers culture slide and allowed to adhere overnight. After treatment, the cells were fixed with 4% paraformaldehyde at room temperature for 10 min. The cells were washed thrice with PBS and permeabilized with 0.2% Triton X-100 for 1 min. Then, the cells were blocked in 3% FBS (diluted in 1% BSA in PBS) for 30 min and washed thrice with PBS before incubation with the primary antibody (rabbit anti-human NF-κB p65) at 4°C overnight. Next, the cells were washed thrice with PBS and incubated for 1 h at room temperature in the dark with the goat anti-rabbit IgG H&L (Alexa Fluor^®^ 488) secondary antibody. The slides were examined using a LEICA DM6000 B inverted fluorescent microscope (Leica, Wetzlar, Germany)

### ELISA

A549 cells (1 × 10^6^ cells) were cultured in a 6-well plate and incubated at 37°C for 24 h in a humidified incubator. Next, the cells were cultured under the conditions of the five experimental groups, and the medium was removed and stored at −80°C until assay. Commercial ELISA kits were used to measure the expression levels of pro-inflammatory cytokines, including TNF-α, IL-1β, IL-6, and IL-8, in the medium, following the manufacturer’s instructions.

### Statistical Analysis

GraphPad Prism 9 for macOS (V 9.2.0; GraphPad Software, San Diego, CA, USA) was used to analyze the data. The data are presented as the mean ± standard deviation (SD). We compared the parameters of the groups using one-way analysis of variance followed by Tukey’s multiple comparison test. Statistical significance was defined as *p* < 0.05.

## Results

### Effects of LPS and AEDS on Cell Viability and ER Stress in A549 Cells

The time courses of LPS responses are shown in **Figures 1A, B**. A549 cells were treated with LPS (50 μg/mL) for 0, 2, 4, 6, 8, 16, 24, and 48 h. The CCK-8 assay results showed a significant decreased cell viability after LPS induction for 8, 16, 24, and 48 h (*p* < 0.001; **Figure 1A**). The Western blotting analysis showed significantly increased expression levels of BiP after LPS induction for 16, 24, and 48 h (*p* < 0.05; **Figure 1B**). The results indicated that LPS induced toxicity and elevated ER stress in the A549 cells.

The dose responses of AEDS are shown in **Figures 1C, D**. A549 cells were treated with AEDS (0, 50, 100, 150, and 200 μg/mL) for 1 h. CCK-8 assay results showed no change in the cell viability after AEDS induction (**Figure 1C**). Western blotting analysis showed significantly decreased expression levels of BiP after AEDS induction at concentrations of 150 and 200 μg/mL for 1 h (*p* < 0.01; **Figure 1D**). The results revealed that AEDS induction had no obvious toxicity and attenuated ER stress in the A549 cells. Based on the previous results, we established experimental protocols with five experimental groups: control, LPS, AEDS, preAEDS, and postAEDS groups (**Figure 1E**).

### AEDS Attenuates ER Stress by Regulating the Proteasomal Degradation to Alleviate ER Stress in A549 Cells

**Figure 2:**
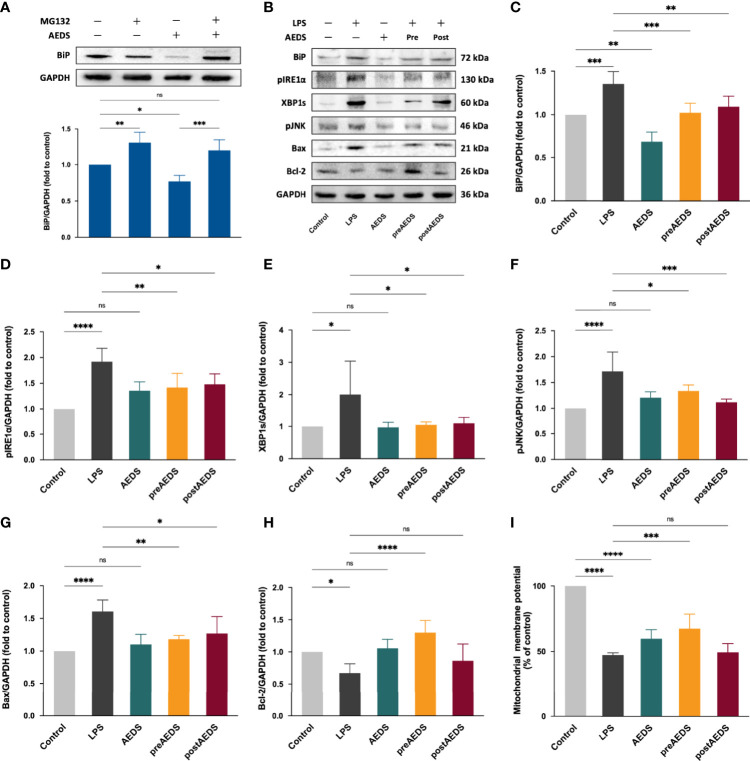
AEDS decreases ER stress by regulating the proteasomal degradation and attenuates LPS-induced ER stress and inositol-requiring enzyme 1 (IRE1)-α-dependent apoptotic unfolded protein response (UPR) cascades in A549 cells. **(A)** Effects of MG132 (10 µM, 6 h), AEDS (200 ug/mL, 1 h), and MG132 + AEDS on binding immunoglobulin protein (BiP) expression in A549 cells. **(B)** Western blotting results of ER stress and IRE1α-dependent apoptotic UPR cascades. **(C–H)** Quantitative Western blotting results of ER stress and IRE1α-dependent apoptotic UPR cascades. **(I)** Mitochondrial membrane potential (MtMP) assay results. MtMP was measured using the JC-1 mitochondrial membrane potential assay kit. All data are expressed as the mean ± SD. All p-values were calculated using one-way analysis of variance followed by Tukey’s *post-hoc* test. The experiment was performed five times in duplicate. *p < 0.05, **p < 0.01, ***p < 0.001, ****p < 0.0001. ns, not significant.

Western blotting results of AEDS and MG132 induction in A549 cells are presented in **Figure 2A**. The results revealed that MG132 significantly increased the expression levels of BiP (*p* < 0.01); while AEDS significantly decreased the expression levels of BiP (*p* < 0.05) compared with the control condition. The results also revealed that MG132 + AEDS significantly increased the expression levels of BiP (*p* < 0.01) compared with the AEDS induction. These results indicated that AEDS attenuates ER stress by regulating the proteasomal degradation to alleviate ER stress.

### AEDS Attenuates LPS-Induced ER Stress and IRE1α-Dependent Apoptotic UPR Cascades in A549 Cells

Western blotting results of BiP, pIRE1α, XBP1s, pJNK, Bax, and Bcl-2 in A549 cells from the five experimental groups are shown in **Figure 2B**. The results showed significantly increased expression levels of BiP, pIRE1α, XBP1s, pJNK, and Bax in the LPS group compared to those in the control group (*p* < 0.05; **Figures 2C–G**). The results showed significantly decreased expression levels of Bcl-2 in the LPS group compared to those in the control group (*p* < 0.05; **Figure 2H**). The results also showed significantly decreased expression of BiP, pIRE1α, XBP1s, pJNK, and Bax in the preAEDS and postAEDS groups compared to those in the LPS group (*p* < 0.05; **Figures 2C–G**). The results showed significantly increased expression of Bcl-2 in the preAEDS group compared to those in the LPS group (*p* < 0.0001; **Figure 2H**). These results indicated that AEDS attenuates LPS-induced ER stress and IRE1α-dependent apoptotic UPR cascades in the A549 cells. We also performed Western blotting analysis on the downstream transcription factors of PERK (ATF4) and ATF6 (ATF6 fragment [ATF6f]), which accounts for the UPR signaling cascades. The results showed no significant differences in the expression levels of ATF4 and ATF6f among the five experimental groups (**Supplementary Figure 1**). The results revealed that LPS and AEDS doesn’t activate the PERK- and ATF6-dependent UPR pathways.

### AEDS Attenuates LPS-Induced MtMP Decrease in in A549 Cells

The results of JC-1 MtMP analysis are presented in **Figure 2I**. The results revealed significantly decreased MtMP in the LPS group (46.99 ± 1.74% of the control group) and AEDS groups (59.51 ± 6.93%) compared to that in the control group (100%; *p* < 0.0001). The results showed significantly increased MtMP in the preAEDS group (67.22 ± 11.17%) compared to the LPS group (46.99 ± 0.71%; *p* < 0.001). These results indicated that AEDS is involved in the regulation of mitochondrial function and exerts protective effects on MtMP during LPS stimulation.

### AEDS Attenuates LPS-Induced NF-κB Nuclear Translocation in A549 Cells

**Figure 3:**
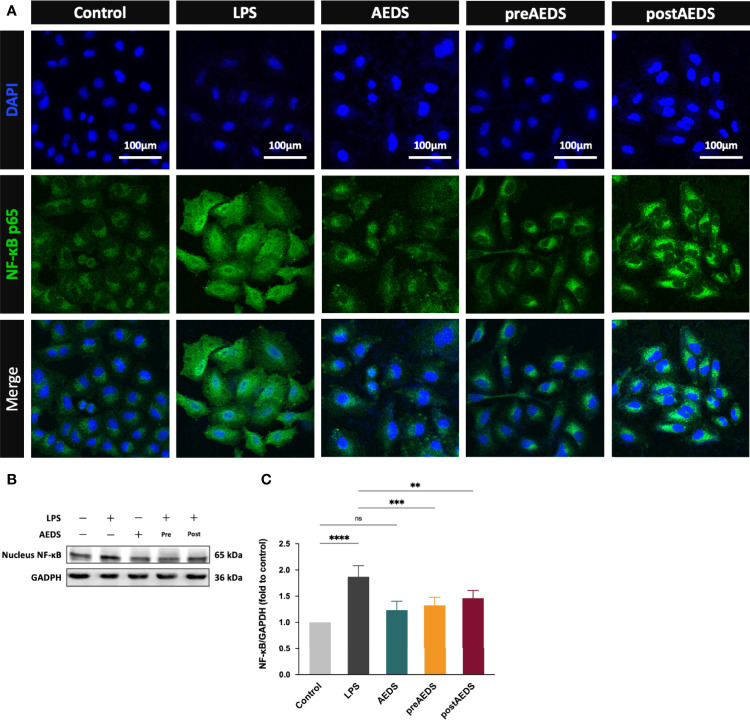
AEDS attenuates LPS-induced nuclear factor-kappa B (NF-κB) nuclear translocation and inflammation in A549 cells. **(A)** Microphotographs (200X) of immunofluorescence analysis of NF-κB p65 and 4’,6-diamidino-2-phenylindole dihydrochloride (DAPI). **(B)** Western blotting results of nucleus NF-κB. **(C)** Quantitative Western blotting results of nucleus NF-κB. All p-values were calculated using one-way analysis of variance followed by Tukey’s *post-hoc* test. The experiment was performed five times in duplicate. **p < 0.01, ***p < 0.001, ****p < 0.0001. ns, not significant.

IF results for 4’,6-diamidino-2-phenylindole dihydrochloride (DAPI) and NF-κB p65 are shown in **Figure 3A**. The results showed significantly increased expression of NF-κB p65 along with obvious NF-κB p65 nuclear translocation in the LPS group compared to those in the control group. IF results showed no obvious differences in the expression of NF-κB p65 in the AEDS group compared to those in the control group. It revealed that AEDS did not induce NF-κB p65 expression in A549 cells. IF results also showed slightly increased expression of NF-κB p65 in the preAEDS and postAEDS groups than in the control group. This suggested the slight activation of the NF-κB signaling pathway in the preAEDS and postAEDS groups. However, the nuclear DAPI signal remained intact, indicating no NF-κB p65 nuclear translocation in the preAEDS and postAEDS groups.

Western blotting results for nucleus NF-κB are presented in **Figures 3B, C**. The results showed significantly increased expression levels of nucleus NF-κB in the LPS group (*p* < 0.0001) compared to those in the control group (**Figure 3C**). The results also showed significantly decreased expression levels of nucleus NF-κB in the preAEDS (*p* < 0.001) and postAEDS (*p* < 0.01) groups compared to those in the LPS group (**Figure 3C**). These results suggested that AEDS attenuates LPS-induced NF-κB nuclear translocation in A549 cells.

### AEDS Attenuates LPS-Induced Inflammation in A549 Cells

**Figure 4:**
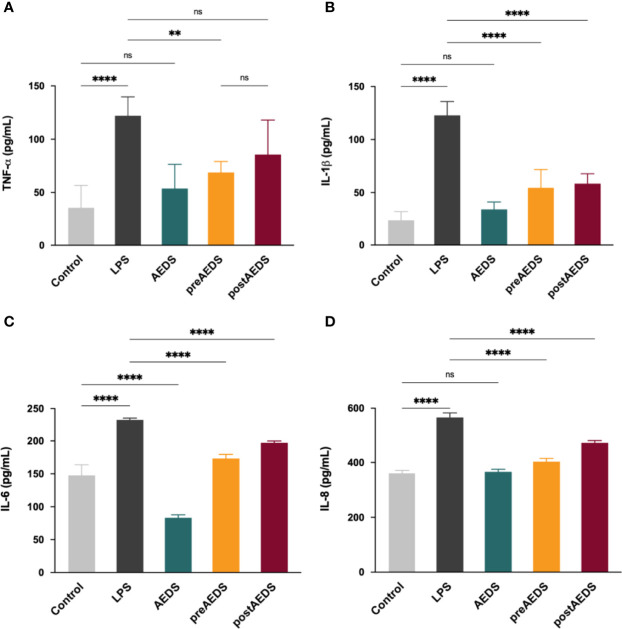
AEDS attenuates LPS-induced elevation in pro-inflammatory cytokine levels. ELISA results of **(A)** tumor necrosis factor (TNF)-α **(B)** interleukin (IL)-1β, **(C)** IL-6, and **(D)** IL-8 levels in the culture medium. All p-values were calculated using one-way analysis of variance followed by Tukey’s *post-hoc* test. The experiment was performed five times in duplicate. **p < 0.01, ****p < 0.0001. ns, not significant.

ELISA results showed significantly increased levels of TNF-α, IL-1β, IL-6, and IL-8 in the LPS group compared to those in the control group (*p* < 0.0001; **Figures 4A–D**). The results showed significantly decreased expression levels of TNF-α, IL-1β, IL-6, and IL-8 in the preAEDS group compared to those in the LPS group (*p* < 0.01; **Figures 4A–D**). The results also showed significantly decreased levels of IL-1β, IL-6, and IL-8 in the postAEDS group compared to those in the LPS group (*p* < 0.05; **Figures 4A–C**). Notably, AEDS induction significantly reduced the levels of IL-6 in the AEDS group compared to those in the control group (*p* < 0.0001; **Figure 4D**). These results indicated that AEDS attenuates LPS-induced inflammation in A549 cells.

### AEDS Attenuates LPS-Induced Apoptotic UPR and Apoptosis in A549 Cells

**Figure 5:**
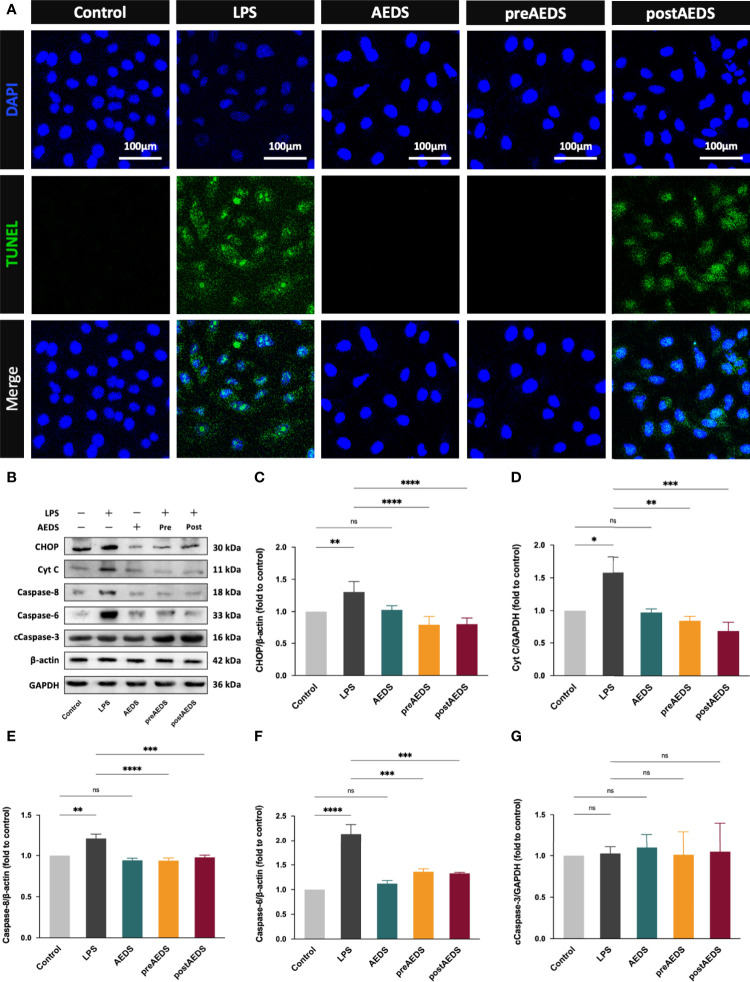
AEDS attenuates LPS-induced apoptotic UPR and apoptosis in A549 cells **(A)** Microphotographs (200X) of immunofluorescence analysis of terminal deoxynucleotidyl transferase dUTP nick and labeling (TUNEL) and DAPI. **(B)** Western blotting results of CCAAT-enhancer-binding protein homologous protein (CHOP), cytochrome c (Cyt C), caspase-8, caspase-6, and cleaved caspase-3 (cCaspase-3). **(C–G)** Quantitative Western blotting results of CHOP, Cyt C, caspase-8, caspase-6, and cCaspase-3. All p-values were calculated using one-way analysis of variance followed by Tukey’s *post-hoc* test. The experiment was performed five times in duplicate. *p < 0.05, **p < 0.01, ***p < 0.001, ****p < 0.0001. ns, not significant.

The results of the TUNEL assay are shown in **Figure 5A**. The results showed significantly increased TUNEL expression levels with apoptotic bodies in the LPS group compared to the control group. These results indicated that LPS induction led to DNA fragmentation in the A549 cells. The results showed no noticeable difference in the expression levels of TUNEL in the AEDS and preAEDS groups compared to those in the control group. This revealed that AEDS did not induce DNA fragmentation in the A549 cells. Moreover, pre-treatment of AEDS significantly prevented LPS-induced DNA fragmentation in the A549 cells. These results also showed significantly decreased TUNEL expression levels of in the postAEDS group compared to those in the LPS group. These results indicated that AEDS exerts protective effects against LPS-induced DNA fragmentation in the A549 cells.

Western blotting results are presented in **Figure 5B**. These results showed significantly increased expression levels of CHOP, Cyt C, caspase-8, and caspase-6 in the LPS group compared to those in the control group (*p* < 0.05; **Figures 5C–F**). The results showed significantly decreased expression levels of CHOP, Cyt C, caspase-8, and caspase-6 in the preAEDS and postAEDS groups compared to those in the LPS group (*p* < 0.01; **Figures 5C–F**). These results also revealed no significant differences in the expression levels of cCaspase-3 among the five experimental groups (**Figure 5G**). These results indicated that AEDS attenuates LPS-induced apoptotic UPR and apoptosis in the A549 cells.

## Discussion

This study demonstrated that AEDS attenuates LPS-induced inflammation and apoptosis by regulating the proteasomal degradation, promoting IRE1α-dependent adaptive UPR, and inhibiting IRE1α-dependent apoptotic UPR (**Figure 5**). The results revealed (1) the effects of AEDS on proteasomal degradation and UPR in enhancing the adaptive ability against ER stress in A549 cells and (2) the pivotal role of IRE1α-dependent UPR in the molecular mechanisms of LPS-induced ALI. These results indicate that AEDS is a potential therapeutic candidate for the treatment of ALI.

ERAD maintains ER protein folding homeostasis and contributes to adaptive UPR (5). A key component of the ERAD pathway, the ubiquitin-proteasome system is responsible for more than 80% of protein degradation in cells (22, 23). Sine AEDS attenuates ER stress without any stimuli (**Figure 1D**), we hypothesized that AEDS exerts its attenuation effects by regulating the ERAD pathway. We used MG132, a proteasome inhibitor, to confirm whether AEDS attenuates ER stress *via* the proteasomal degradation pathway (21). We found that AEDS attenuated ER stress by regulating the proteasomal degradation and promoting adaptive UPR to alleviate ER stress (**Figure 2A**). As ER stress and UPR are involved in ALI and other pulmonary disorders (24, 25), upregulating the adaptive ability of pulmonary cells could be used as a potential treatment strategy. Our results indicate that AEDS enhances the adaptive ability against ER stress, with or without LPS stimulation, by regulating the proteasomal degradation in lung epithelial A549 cells. These results also demonstrated the significant protective effects of AEDS against inflammation and apoptosis during LPS stimulation, which further highlights its potential for the treatment of pulmonary disorders.

One of the most critical interactions between ER and mitochondria is calcium signaling between the two organelles (7). Inositol 1,4,5-trisphosphate receptors (IP3Rs) clustered in the mitochondria-associated ER membranes regions primarily account for the Ca^2+^ transportation from ER. Bcl-2 inhibits IP3Rs and Ca^2+^ transportation from the ER (26). During apoptotic UPR, the downregulated antiapoptotic Bcl-2 and upregulated pro-apoptotic Bax and Bak release the Ca^2+^ from ER into mitochondria (27).BiP prolongs calcium signaling from the ER to mitochondria by stabilizing IP3R at the mitochondria-associated membranes (7). The excessive Ca^2+^ influx leads to mitochondrial dysfunction and apoptosis (27). Since our results showed that AEDS attenuated LPS-induced decreased expression levels of Bcl-2 in the A549 cells, we performed a JC-1 MtMP assay to investigate the effects of LPS and AEDS on MtMP changes. The results showed that LPS or AEDS induction decreased MtMP in the AEDS group compared to those in the control group. The results showed a significant increase in MtMP in the preAEDS group compared to that in the LPS group. Overall, the results revealed that AEDS is involved in the regulation of mitochondrial function and shows relative protective effects on MtMP during LPS stimulation. Various components of AEDS may affect the mitochondrial function *via* multiple pathways (14).

Among the three ER transmembrane sensors, IRE1α is recognized as an administrator of cell fate (6). IRE1α is characterized by its RNase and kinase domains, which allow it to activate either the adaptive or apoptotic UPR cascades (28). IRE1α signaling persists during ER stress and triggers different UPRs according to its requirements (6). In the adaptive phase, IRE1α-mediated *Xbp-1* mRNA splicing activates adaptive UPR target genes. In the transition phase, the signaling increases ER stress *via* mRNA decay of selective UPR target genes. In the apoptotic phase, cell death is initiated by the activation of *IRE1α–Casp2* signaling (6). Schmitz et al. indicated that the NF-κB pathway is primarily activated by cytokines or Toll-like receptor (TLR) agonists rather than ER stress during infection or inflammation (29). However, the effects of ER stress and UPR regulation were demonstrated in LPS-induced ALI models (10–12, 30). Moreover, the present study revealed the pivotal role of IRE1α-dependent UPR that activates diverse pathways, including the NF-κB pathway, in LPS-induced ALI in the A549 cells. We suggest that, in addition to the TLR cascades, the UPRs, especially IRE1α-dependent UPR, also plays an essential role in infection or inflammation in ALI.

ER stress not only affects the pathological status but is also important for healthy cell protein homeostasis (5). Meanwhile, UPR is a relative upstream regulatory pathway facing endogenous and exogenous stimuli (5). We proposed that IRE1α-dependent UPR is also important in other kinds of ALI, even in other lung diseases. Pao et al. reported that 4-PBA decreased the hyperoxia-induced up-regulation of BiP, PERK, IRE1α, ATF4, ATF6, eIF2, and CHOP expression levels as well as the inflammation and apoptosis pathways in both MLE-12 cells and C57BL/6J mice (31). The results indicated that IRE1α also participates in hyperoxia-induced ALI. Disturbance of UPR, including the IRE1α cascade, is reported to drive the development of pulmonary disorders, such as cigarette smoke exposure, pulmonary infection, pulmonary fibrosis, asthma, cystic fibrosis, and lung cancer (24). Based on our results, AEDS and the IRE1α-dependent UPR should be further investigated in various models of ALI and other pulmonary disorders.

## Limitation of the Study

The current study has several limitations. First, we performed this study using human lung epithelial A549 cells. We demonstrated that AEDS is effective in attenuating LPS-induced A549 cell injuries. We also demonstrated the protective mechanisms of AEDS *via* the UPR. However, further clinical studies are necessary to confirm the effects of AEDS in humans. Second, the experiments were conducted during the acute stages of ALI; therefore, further studies on the long-term effects of AEDS are necessary. Third, the entire AEDS was used in our experiments. So far, approximately 67 compounds have been identified in *Descurainia Sophia* (32). Among the identified phytochemical constituents of DS, coumarins, flavonoids, and lignan are the possible active ingredients that exert protective effects on LPS-induced ALI in A549 cells, with respect to their anti-inflammatory effects and the water solubility (13). Therefore, more rigorous pharmaceutical analyses are needed to identify the specific components that regulate the proteasomal degradation and IRE1α-dependent UPR.

## Conclusions

**Figure 6:**
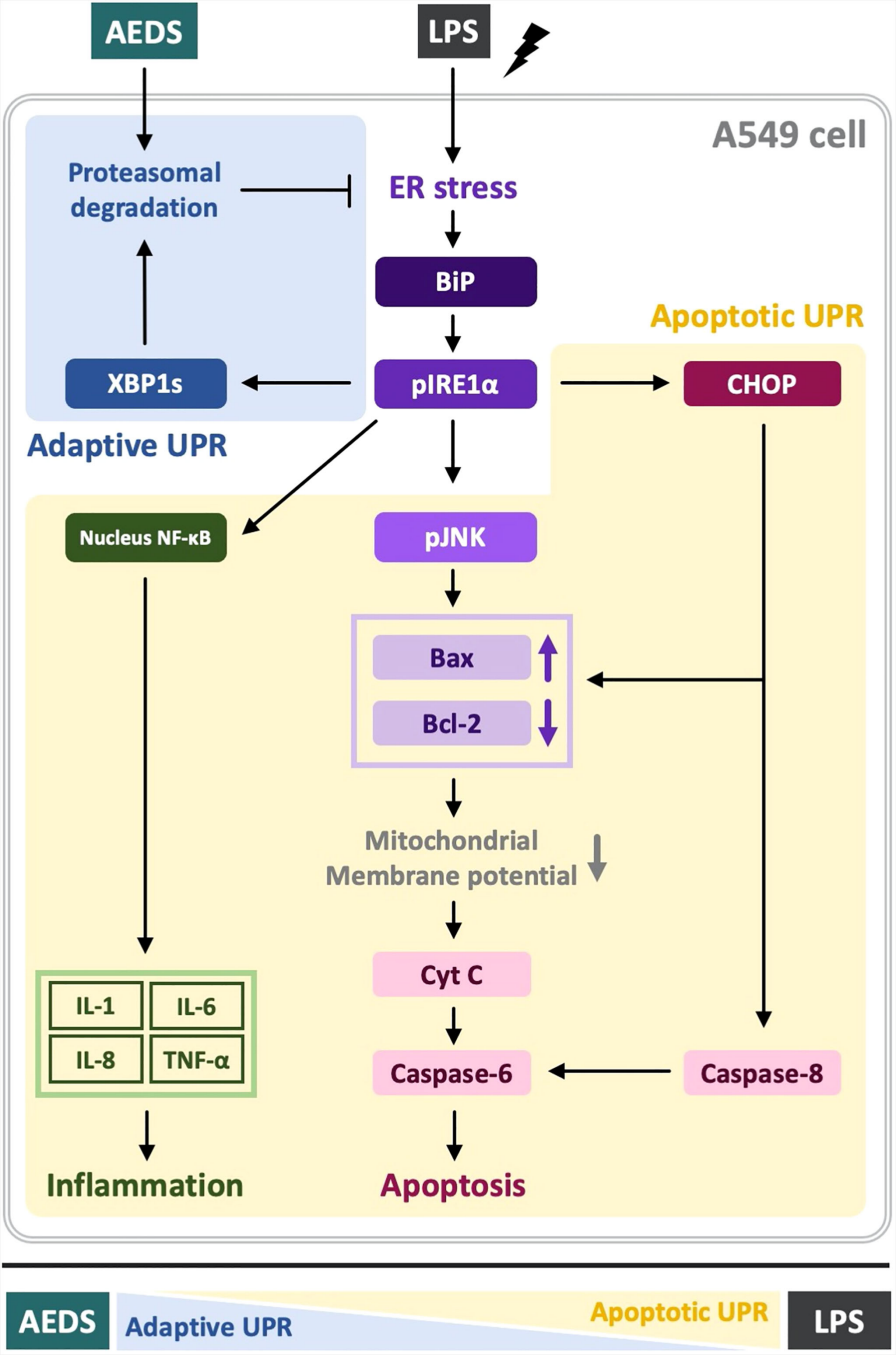
Mechanisms of LPS-induced acute lung injury (ALI) and protective role of AEDS. AEDS decreases LPS-induced inflammation and apoptosis by regulating the proteasomal degradation, promoting IRE1α-dependent adaptive UPR, and inhibiting IRE1α-dependent apoptotic UPR. IRE1α-dependent UPR plays a pivotal role in LPS-induced ALI. AEDS tends to induce adaptive UPR, whereas LPS induces apoptotic UPR.

AEDS attenuates LPS-induced inflammation and apoptosis by regulating the proteasomal degradation, promoting IRE1α-dependent adaptive UPR, and inhibiting IRE1α-dependent apoptotic UPR. Moreover, IRE1α-dependent UPR plays a pivotal role in LPS-induced ALI. AEDS tends to induce adaptive UPR, whereas LPS induces apoptotic UPR (**Figure 6**). Therefore, AEDS can be used as a potential therapeutic candidate for the treatment of patients with ALI.

## Data Availability Statement

The original contributions presented in the study are included in the article/**Supplementary Material**. Further inquiries can be directed to the corresponding authors.

## Author Contributions

The authors confirm contribution to the paper as follows: study conception and design: P-CH, C-KP, C-CL, K-LH; data collection: P-CH, G-TL, I-ST, C-YK; statistical analysis: P-CH, G-TL, I-ST, C-YK; interpretation of results: P-CH, C-KP, M-CW, C-CL, K-LH; drafting manuscript: P-CH; project administration: C-CL, K-LH. All authors reviewed the results and approved the final version of the manuscript.

## Funding

This work was supported by the Buddhist Tzu Chi Medical Foundation, Taiwan [TCMF-CM2-111-04 and TCMF-CP 109-02(110)], and the Taipei Tzu Chi Hospital, Buddhist Tzu Chi Medical Foundation, New Taipei City, Taiwan [TCRD-TPE-110-44].

## Conflict of Interest

The authors declare that the research was conducted in the absence of any commercial or financial relationships that could be construed as a potential conflict of interest.

## Publisher’s Note

All claims expressed in this article are solely those of the authors and do not necessarily represent those of their affiliated organizations, or those of the publisher, the editors and the reviewers. Any product that may be evaluated in this article, or claim that may be made by its manufacturer, is not guaranteed or endorsed by the publisher.
