# Supplementary material for: Aqueous Extract of Descuraniae Semen Attenuates Lipopolysaccharide-Induced Inflammation and Apoptosis by Regulating the Proteasomal Degradation and IRE1α-Dependent Unfolded Protein Response in A549 Cells
Source: Front Immunol. 2022 Jun 24;13:916102. doi: 10.3389/fimmu.2022.916102 (PMC9265213; doi:10.3389/fimmu.2022.916102)

Supplementary Figure 1

(A) Western blotting results of activating transcription factor 4 (ATF4). (B) Western blotting results of activating transcription factor 6 fragment (ATF6f). All p-values were calculated using one-way analysis of variance followed by Tukey's post-hoc test. The experiment was performed three times in duplicate.

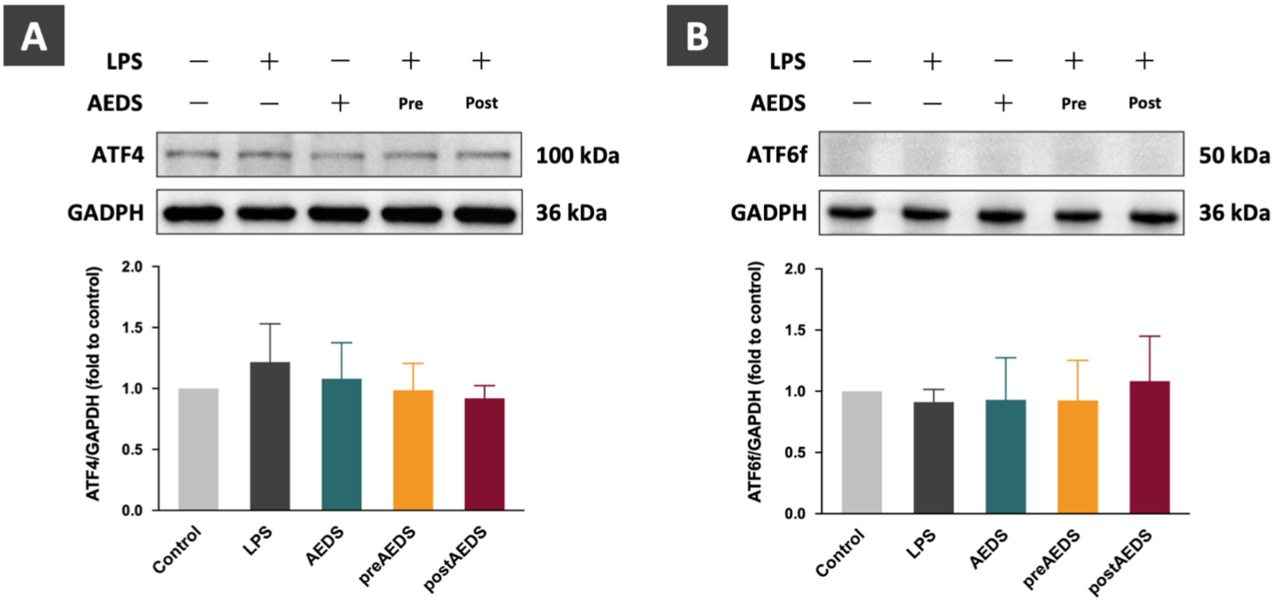

Supplement: Supplementary file 1 [file Image_1.pdf]
